# Explaining the abundance of distant analogies in naturalistic observations of experts

**DOI:** 10.3389/fpsyg.2014.01487

**Published:** 2014-12-22

**Authors:** Máximo Trench

**Affiliations:** Psychology Department, Centro Regional Universitario Bariloche, National University of ComahueBariloche, Argentina

**Keywords:** analogy, naturalistic settings, experts, surface similarty, retrieval

Analogical reasoning is a landmark of human cognition. Based on the realization that the elements of two situations are organized by similar systems of relations, analogical inferences allow the transfer of knowledge structures from a better-known situation (the base analog) to a target situation that is relatively less understood (the target analog).

Experimental research has demonstrated that the retrieval of base analogs from long term memory in response to the proceesing of a target analog is infrequent in the lack of semantic similarities between both situations (Gick and Holyoak, 1980; Keane, 1987; Gentner et al., 1993; Trench and Minervino, 2014). With the turn of the century, several naturalistic observations of experts working in their domains of expertise yielded a more complex picture. While molecular biologists (Dunbar, 1997) and psychologists (Saner and Schunn, 1999) still exhibited mostly within-domain analogizing, the observation of journalists and politicians (Blanchette and Dunbar, 2001), teachers (Richland et al., 2004), managers (Bearman et al., 2007) and design engineers (Christensen and Schunn, 2007) showed a more frequent use of long-distance analogies. The naturalistic study by Kretz and Krawczyk (2014) on the use of analogies by economists also demonstrates an abundance of distant analogies in the sevice of an impressive variety of communicative purposes, most of which were not evident in prior research. These goals included the generation of concrete source examples of more general target concepts, the formation of visual images of source concepts, the addition of colorful speech, the inclusion of a target into a source concept, or the differentiation between source and target concepts. With these results in mind, the time is ripe to assert that the naturalistic observation of experts shows a more flexible use of analogical sources than is predicted by experimental studies on analogical transfer, and simulated by dominant computer models of analogical retrieval (e.g., MAC/FAC, Forbus et al., 1995; LISA, Hummel and Holyoak, 1997). How, then, to explain this analogical abundance? In trying to account for the contrasting results of the experimental and the naturalistic traditions, the default explanations revolve around the expertise of the analogizers and the psychological constraints of the target tasks. I will argue that although both factors are likely to bear some responsibility for this empirical inconsistency, there are reasons to expect a heavier weight of the latter.

## The expertise of the analogizers

Shortly after having documented that journalists and politicians generated mostly distant analogies when arguing for (or against) the referendum on the independence of Quebec, Blanchette and Dunbar (2000) obtained an *in vitro* replication of this result with novice participants generating their own analogies for another realistic political topic: the zero-deficit strategy for controlling public debt. The authors concluded that their prior results were due to the fact that the analogizers were generating their own analogies for a realistic situation, rather than to their expertise in the target issue. Trench et al. (2009a) provided support for this interpretation by replicating Blanchette and Dunbar's results with 10 different target topics. In the same vein, Bearman et al. (2007) failed to observe differences in the analogies proposed by novices and experts solving management problems. Rather than based on broad expertise differences across-participants, it seems that the ease of generating distant analogies depends on the goals of the analogizer and on the extent to which she understands the target analog at stake. When the analogizer comprehends the target analog better than her intended audience, as in communicative situations such as explaining a procedure to students (Richland et al., 2004) or selling political ideas to the population (Blanchette and Dunbar, 2000, 2001; Trench et al., 2009a), both experts and novices easily generate distant analogies. But when the target analog is insufficiently understood, as when Dunbar's (1997) expert molecular biologists or Gick and Holyoak's (1980) novice participants are attempting to solve a problem, distant analogies are seldom generated.

## Psychological constraints of the target task

Another explanation for the frequent use of distant analogies in naturalistic studies might arise from comparing the psychological constraints of naturalistic analogy generation against those of classical experimental studies. The standard experimental procedure comprises an encoding phase, during which participants learn the base analogs, and a transfer phase, where experimenters present participants with either a semantically close or a semanically distant target situation, and assess whether its processing elicits the spontaneous retrieval of the base analog. Based on this procedure, differences accross conditions were typically taken to demonstrate the centrality of surface similarities during analogical transfer. In contrast with this highly controlled environment—in which transfer can only originate in the retrieval of the critical base analog from memory—in naturalistic settings participants are free to generate analogies by means of retrieving their own base analogs (Blanchette and Dunbar, 2000; Hofstadter and Sander, 2013), identifying conceptual metaphors (Minervino et al., 2009), stumbling across suitable analogs in the external environment (Christensen and Schunn, 2005) or fabricating novel base analogs either by generating extreme cases out of the target analog (Clement, 1988), or by reinstantiating the relational structure of the target with a new set of elements (Olguín et al., 2013). Upon generating candidate analogies via any combination of such mechanisms, the proportion of close vs. distant analogies that people produce in naturalistic settings can also reflect a conscious editing of one type of analogies in favor of the other type, depending on the purpose of the reasoner (Trench et al., 2009b).

The fact that the core components of our retrieval mechanisms are invariably set to favor semantically close base analogs (Gentner et al., 1993; Trench and Minervino, 2014) suggests that the abovementioned generative mechanisms could possibly account for the frequency and the diversity of the analogies generated by experts. Future studies, both naturalistic and experimental, are required to understand how these overlooked analogy generation methods interact with the variety of goals that realistic analogy generation can pursue, as eloquently revealed by Kretz and Krawczyk's (2014) detailed analysis of the analogies produced by expert economists.

## Conflict of interest statement

The author declares that the research was conducted in the absence of any commercial or financial relationships that could be construed as a potential conflict of interest.
